# Is the relationship of body mass index to severity of coronary artery disease different from that of waist-to- hip ratio and severity of coronary artery disease? Paradoxical findings

**DOI:** 10.5830/CVJA-2014-054

**Published:** 2015

**Authors:** Amir Farhang Zand Parsa, Bahareh Jahanshahi

**Affiliations:** Division of Cardiology, Imam Khomeini Hospital Complex, Tehran University of Medical Sciences, Tehran, Iran; Division of Cardiology, Imam Khomeini Hospital Complex, Tehran University of Medical Sciences, Tehran, Iran

**Keywords:** body mass index, waist-to-hip ratio, coronary artery disease, SYNTAX score, Duke score

## Abstract

**Background:**

Although for decades there has been controversy regarding the relationship between obesity and coronary artery disease (CAD), it has been assumed that high body mass index (BMI) is a risk factor for CAD. However, the findings of some recent studies were paradoxical.

**Objectives:**

The aim of this study was to find a relationship between high BMI and waist-to-hip ratio (WHR) with severity of CAD.

**Methods:**

This study was a cross-sectional, prospective study where 414 patients with suspected coronary artery disease, in whom coronary angiography was performed, were enrolled. The mean ± SD of their ages was 61.2 ± 27.4 years (range 25–84), and 250 (60.4%) were male. Regarding cardiovascular risk factors, 113 (27.3%) patients had a history of diabetes mellitus (DM), 162 (39.1%) had hypercholesterolaemia, 238 (57.4%) had hypertension, 109 (26.3%) were current smokers and 24 (5.8%) had a family history of CAD. The mean ± SD of the patients’ BMI was 26.04 ± 4.08 kg/m^2^ (range 16–39) and means ± SD of their WHR ranged from 0.951 ± 0.07 to 0.987 ± 0.05. The mean ± SD of the severity of CAD according to the SYNTAX and Duke scores were 17.7 ± 9.6 (range 0–64) and 3.2 ± 1.7 (range 0–12), respectively.

**Results:**

In this study, findings showed a negative correlation between the severity of CAD and BMI, according to both SYNTAX and Duke scores (*p* ≤ 0.001 and *p* = 0.001, respectively). However, there was a positive correlation between WHR and severity of CAD, according to the Duke score (*p* = 0.03).

**Conclusion:**

BMI had a negative correlation with the severity of CAD, but waist-to-hip ratio had a positive correlation with severity of CAD.

## Abstract

Although obesity has been regarded as an independent risk factor for coronary artery disease (CAD) by the American Heart Association (AHA) and investigators of the Framingham Heart study in the 1980s and 1990s,^1^-^3^ this has not been supported by recent clinical trials. Moreover, the positive linear relationships between obesity and CAD, as reported by some studies, were as a result of univariate analysis of their data. However, by using multivariate analysis of these study data, which included other cardiovascular risk factors such as diabetes mellitus (DM), hypertension (HTN) and hyperlipidaemia, this relationship was shown to be dramatically reduced.^4^,^5^

In the Munster Heart study (PROCAM) and similar studies, the positive relationship between body mass index (BMI) and cardiovascular risk factors, with cardiac mortality, which attributed obesity as an independent risk factor, appeared to be due to the associated cardiovascular risk factors that usually accompany obesity.^6^-^10^ In these studies there was also a strong positive correlation between high BMI and other cardiovascular risk factors.

However, findings of recent studies in this regard were opposite to those of previous studies. According to their findings, not only was obesity not a risk factor for CAD but it also had a protective effect on the progression of CAD, which is known as the ‘obesity paradox’.^11^,^12^ On the other hand, abdominal adiposity has always been associated with increased cardiovascular disease and mortality rate, independent of patients’ weight.^13^,^14^

This study was designed to evaluate not only the impact of BMI but also waist-to-hip ratio (WHR) on the severity of CAD, based on angiographic findings.

## Methods

This study was a cross sectional, prospective study that was conducted in our hospital from September 2009 to March 2011. A total of 414 patients with suspected CAD were enrolled in the study. Patients’ mean age ± SD was 61.2 ± 27.4 years (range 24–84) and 250 (60.4%) patients were male.

**Fig. 1.**
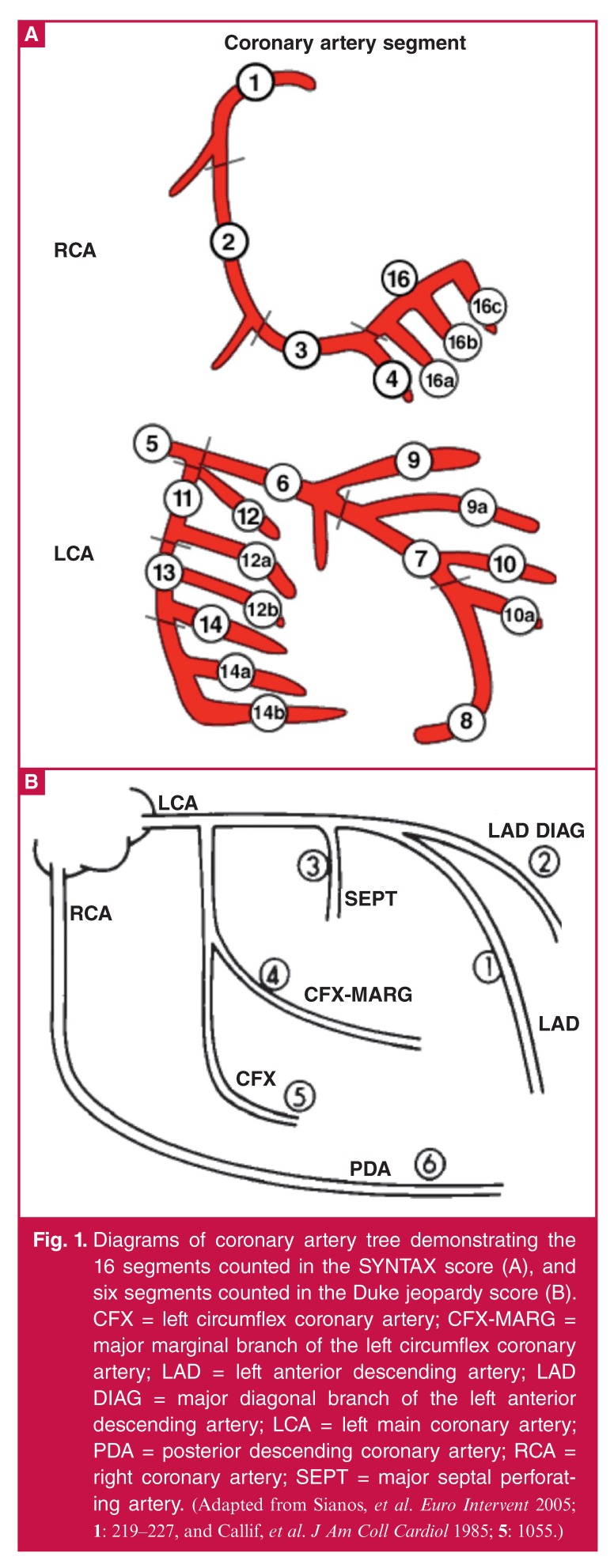
Diagrams of coronary artery tree demonstrating the 16 segments counted in the SYNTAX score (A), and six segments counted in the Duke jeopardy score (B). CFX = left circumflex coronary artery; CFX-MARG = major marginal branch of the left circumflex coronary artery; LAD = left anterior descending artery; LAD DIAG = major diagonal branch of the left anterior descending artery; LCA = left main coronary artery; PDA = posterior descending coronary artery; RCA = right coronary artery; SEPT = major septal perforating artery. (*Adapted from Sianos, et al. Euro Intervent 2005; 1: 219–227, and Callif, et al. J Am Coll Cardiol 1985; 5: 1055*.)

Coronary angiography was done on all patients. The severity of CAD was measured using the SYNTAX score (the sum of the points assigned to each individual lesion identified in the coronary arteries with > 50% stenosis in vessels > 1.5 mm diameter). The SYNTAX score, a lesion-based angiographic scoring system, was introduced as a tool to grade the complexity of CAD. It was derived from a combination of the AHA classification for coronary artery segments with various other scores,^15^,^16^ and the Duke jeopardy scores (Fig. 1A). The Duke jeopardy score is a simple, effective scoring system for quantifying the amount of myocardium at risk. The Duke jeopardy score, developed by Dash *et al.*, 1977,^17^ and validated by Califf *et al.*,1985,^18^ detects the main vessels affected in their large branches, Fig. 1B).

Coronary angiographies of patients were reviewed by two experts who were blinded to the patients’ BMI and WHR. Patients were divided into five groups according to their BMI; normal BMI (21–24 kg/m^2^), overweight (25–29 kg/m^2^), class I obesity (30–34 kg/m^2^), class II obesity (35–39 kg/m^2^) and class III obesity (> 40 kg/m^2^). Also patients were divided into four groups according to their age; 20–39, 40–59, 60–79 and > 80 years old.

Inclusion criteria were patients over 20 years old who had definite indications for coronary angiography, based on their clinical background. The exclusion criteria were patients unwilling to participate in the study.

For the purpose of multivariate analysis, we included in the study evaluations of conventional cardiovascular risk factors, such as HTN (systolic blood pressure ≥ 140 mmHg and/diastolic blood pressure ≥ 90 mmHg), DM [fasting blood sugar > 126 mg/dl (6.99 mmol/l) and/glycosylated haemoglobin (HbA_1c_) > 6%], hyperlipidaemia [low-density lipoprotein (LDL) cholesterol > 120 mg/dl (3.11 mmol/l) and triglycerides > 150 mg/dl (1.7 mmol/l)], family history of CAD and cigarette smoking (current smoker: at least five cigarettes/day for ≥ one year).

## Statistical analysis

For analysing data, SPSS version 15 (USA, Illinois, Chicago) was used. The Student’s *t*-test was used for comparing quantitative variables between two groups and the one-way ANOVA test was used for comparing means of quantitative variables between groups. Logistic regression was used for multivariate analysis of compounding factors. Chi-square and Fisher’s exact tests were used for analysis of qualitative variables and a *p*-value ≥ 0.05 was considered significant.

## Results

Of 414 (100%) patients, 250 (60.4%) were male and their ages ranged from 25 to 84 years. The prevalences of DM, HTN, hyperlipidaemia, family history of CAD and cigarette smoking were 27.3, 29.5, 39.1, 5.8 and 26.3%, respectively. Basic clinical and demographic characteristics of the patients are presented in Table 1.

**Table 1:** Basic clinical and demographic characteristics of patients.

*Characteristics*	*Number (%)*
Age, mean ± SD (years)	61.2 ± 27.4
Male gender	250 (60.4)
Diabetes mellitus	113 (27.3)
Hypertension	122 (29.5)
Hyperlipidaemia	162 (39.1)
History of CAD	24 (5.8)
Cigarette smoking	109 (26.3)
History of AP	254 (85.5)
History of MI	85 (20.5)

CAD = coronary artery disease, AP = angina pectoris, MI = myocardial infarction.

The severity of CAD was measured by the SYNTAX and Duke jeopardy scores. For the SYNTAX score, the mean ± SD of the patients’ scores was 17.7 ± 9.6 (range 0–64) and for the Duke score, it was 3.2 ± 1.7 (range 0–12). There was a negative correlation between the SYNTAX and Duke scores (severity of CAD) and the patients’ BMI (*p* = 0.01 and *p* = 0.001, respectively).The correlation between the patients’ BMI and the severity of CAD (SYNTAX and Duke scores) is presented in Table 2.

**Table 2:** Correlation between BMI and severity of CAD (SYNTA X and Duke scores)

*BMI (kg/m²)*	*Number of patients (%)*	*SYNTAX score (mean ± SD)*	*Duke score (mean ± SD)*
20–24	169 (40.8)	22.3 ± 17.2	4.01 ± 3.3
25–29	154 (37.2)	16.1 ± 14.6	3.05 ± 2.5
30–34	83 (20.1)	12.1 ± 9.2	2.3 ± 1.1
35–39	8 (1.9)	10.8 ± 7.04	1.8 ± 1.04
*p*-value	–	0.01	0.001

BMI = body mass index

There was an inverse relationship between obesity and the severity of CAD, according to the SYNTAX and Duke criteria, which has been defined as the ‘obesity paradox’. In order to rule out the impact of other cardiovascular risk factors, multivariate regression analysis was performed. Regression analysis revealed a β-coefficient of –0.14 for the Duke score and –0.17 for the SYNTAX score. This means that for every unit increase in BMI there would be a 0.14 and 0.17 decrease in the severity of CAD according to the Duke and SYNTAX scores, respectively. After adjusting for confounding factors, there was still a significantly negative correlation between BMI and severity of CAD (*p* = 0.028 and 0.01, respectively). Meanwhile multivariate analysis revealed a positive correlation between severity of CAD and cardiovascular risk factors (Table 3).

**Table 3:** Correlation between cardiovascular risk factors and severity of CAD (Duke and SYNTA X scores)

*Risk factors*	*Duke score (mean ± SD)*	*p-value*	*SYNTAX score (mean ± SD)*	*p-value*
Hypertensives	3.6 ± 1.7	0.04	19.1 ± 13.1	0.03
Normotensives	2.4 ± 1.9		14.9 ± 9.5	
Cigarette smokers	3.8 ± 1.2	0.02	20.8 ± 17.4	0.03
Non-smokers	3.07 ± 1.4		16.6 ± 14.2	
Hyperlipidaemics	3.9 ± 1.5	0.001	31.5 ± 18.05	0.001
Normolipidaemics	2.8 ± 1.2		15.3 ± 11.02	
Diabetics	4.1 ± 3.6	0.002	21.5 ± 18.4	0.008
Non-diabetics	2.9 ± 1.3		16.3 ± 9.2	
FH positive	4.5 ± 3.1	0.07	21.9 ± 14.2	0.3
FH negative	3.1 ± 2.3		17.5 ± 10.4	

FH = family history.

On the other hand, our findings regarding the relationship between WHR and severity of CAD, based on the Duke myocardial jeopardy score, showed a positive correlation between the two variables (*p* = 0.03). With increasing WHR, the Duke score also increased. The relationship between severity of CAD (Duke score) and WHR is presented in Table 4.

**Table 4:** Relation between WHR and severity of CAD based on the Duke score

*WHR (mean ± SD)*	*Number of patients*	*Duke score*
0.951 ± 0.07	165	0
0.954 ± 0.06	62	2
0.957 ± 0.07	58	4
0.962 ± 0.05	54	6
0.971 ± 0.05	44	8
0.979 ± 0.02	24	10
0.987 ± 0.05	6	12
p-value	0.03	

WHR = waist-to-hip ratio.

## Discussion

In this study, there was a paradoxical relationship between BMI and severity of CAD but not between WHR and severity of CAD. Based on the SYNTAX and Duke scores, β-coefficients between BMI and severity of CAD before multivariate analysis were –0.2 and –0.18, respectively. After multivariate analysis, they were –0.17.and –0.14, respectively. This shows an inverse relationship between BMI and severity of CAD.

Controversy regarding the correlation between obesity and CAD, which surfaced a few decades ago, was the motivation for us to conduct this study. Although it seems logical that obesity or adiposity should be accompanied by more accumulation of fat cells everywhere in the body, including vascular walls (atherosclerotic plaques), it must be clarified that first of all, obesity *per se* is not adiposopathy, and second, the process of atherosclerosis is not a simple process of fat accumulation.^19^,^20^

The process of atherosclerosis is inflammation as a result of the response to injury in the milieu of high intravascular LDL cholesterol, especially oxidised LDL. It seems that visceral adipose tissue is metabolically more active and pathological than subcutaneous adipose tissue, and induces immunity processes that contribute to atherosclerotic cardiovascular disease.^21^-^24^ The answer to the question raised from the obesity paradox is that atherosclerotic disease does not result from the accumulation of adipose tissue *per se* but is as a result of adipose tissue dysfunction, or ‘sick fat’.^19^,^23^,^24^

Rubinshtein and colleagues (2006), in their study on 928 patients with CAD, showed that obesity had an inverse relationship with the severity of CAD but other risk factors such as DM, hyperlipidaemia and male gender were correlated with the severity of CAD.^11^ In another study, published in 2007 by Niraj and colleagues, which was similar to our study, the relationship between severity of CAD and BMI according to the Duke score was also paradoxical.^10^ Although there are similarities between our study and theirs regarding the inverse relationship between patients’ BMI and the severity of CAD, in our study the relationship between WHR and severity of CAD was evaluated simultaneously. Surprisingly, in our study, WHR was correlated with the severity of CAD based on the Duke score.

Moreover, according to the studies of Morricone, Empana and Zhang, which were published in 1999, 2004 and 2008, respectively, abdominal adiposity and severity of CAD were correlated.^12^-^14^ Although their findings were similar to ours regarding correlation between WHR/abdominal obesity and severity of CAD, they did not compare BMI with WHR regarding their impact on the severity of CAD, as we did. These studies showed that, first, high BMI per se was not a risk factor for CAD, and second, high WHR/abdominal obesity was a risk factor for CAD. That means abdominal fat accumulation is more pathological (adiposopathic) than subcutaneous fat ccumulation.^19^,^24^

Although in our study, regression analysis for confounding factors such as DM, HTN, cigarette smoking and hyperlipidaemia revealed a statistically significant correlation between them and the severity of CAD (*p* = 0.002, *p* = 0.001, *p* = 0.04 and *p* = 0.02, respectively), after omission of confounding factors, there was still a paradoxical relationship between BMI and severity of CAD. β-coefficients before multivariate analysis were –0.2 and –0.18, and after multivariate analysis they were –0.17 and –0.14, based on the SYNTAX and Duke scores, respectively. This showed an inverse relationship between BMI and severity of CAD.

The limitation of our study was that lower BMI (20–24 kg/m^2^) was more prevalent (56.2%) in the older age groups (> 60 years), and higher BMI (30–34 kg/m^2^) was more common (57.8%) in the younger age groups (40–59 years). As in the study by Niraj *et al.*,^11^ it can be concluded that patents with a higher BMI have been evaluated earlier for CAD. This indicates a need for a larger study with more age-matched groups.

## Conclusion

The findings of this study, paradoxically, showed a negative correlation between BMI and the severity of CAD, but a positive correlation between WHR and the severity of CAD.
